# Occlusion Collusion: A Case of Acute Aortic Occlusion Mistaken for Acute Spinal Infarction

**DOI:** 10.7759/cureus.85491

**Published:** 2025-06-07

**Authors:** Rachel E Bridwell, Robert Conrad, Angela Curell, Joseph M Fierro, Brit Long

**Affiliations:** 1 Emergency Medicine, Atrium Health, Charlotte, USA; 2 Trauma Surgery, Atrium Health, Charlotte, USA; 3 Anesthesiology, University of Cincinnati Medical Center, Cincinnati, USA; 4 Emergency Medicine, Brooke Army Medical Center, Fort Sam Houston, USA

**Keywords:** acute aortic occlusion, atrial fibrillation, back pain, lower extremity weakness, saddle aortic embolism

## Abstract

Acute aortic occlusion (AAO) carries high morbidity and mortality, though it can present with vague symptoms that mimic other less serious pathologies. Various comorbidities will predispose patients to AAO, including any hypercoagulable conditions, hypertension, diabetes, and tobacco use. The level of occlusion dictates symptomatology, with distal occlusions frequently mimicking neurologic pathology. Prompt imaging and vascular surgery consultation are critical for definitive management. Due to the challenging and protean presentations and severe complications, emergency clinicians must maintain a high index of suspicion for an AAO.

## Introduction

Acute aortic occlusion (AAO) is a rare but serious diagnosis, fraught with diagnostic mimicry and significant morbidity and mortality. Occurring secondary to sudden embolic or thrombotic phenomena, AAO most commonly occurs in patients between 60 and 70 years old and may present in a myriad of ways depending on the level of occlusion and tissues affected (e.g., gastrointestinal, lower extremities, kidneys) [1, 2]. Thromboemboli can arise from atrial fibrillation as well as the proximal aorta, or in situ, or can occur from direct atherosclerosis of the aorta. Other risk factors include hypertension, tobacco use, diabetes mellitus, history of cancer, chronic kidney disease, pulmonary disease, and inherited coagulopathy [3]. There are a variety of levels that may be affected, which impacts the presentation; infrarenal lesions often present with vague symptoms including low back pain, sensory changes, and lower extremity pain or weakness akin to the presentation of spinal myelopathy, transverse myelitis, or other aortic disease [4]. The authors present a rare case of acute onset lower extremity weakness following a mechanical ground-level fall in an 84-year-old female patient, thought to have acute spinal infarction, but instead found to have an infrarenal AAO.

## Case presentation

An 84-year-old female patient presented to a level 1 trauma center emergency department (ED) following a ground-level fall with complaints of lower extremity weakness and sensory changes. Her past medical history was pertinent for hypertension, atrial fibrillation on apixaban, dyslipidemia, prior cerebrovascular event, heparin-induced thrombocytopenia, and right popliteal artery embolus status post thrombectomy. She additionally noted hitting her head, but she denied loss of consciousness. She also denied any preceding neurologic complaints, chest pain, palpitations, and dyspnea. She endorsed previous tobacco use but stopped smoking 10 years prior with a 40-pack-year history. 

**Figure 1:**
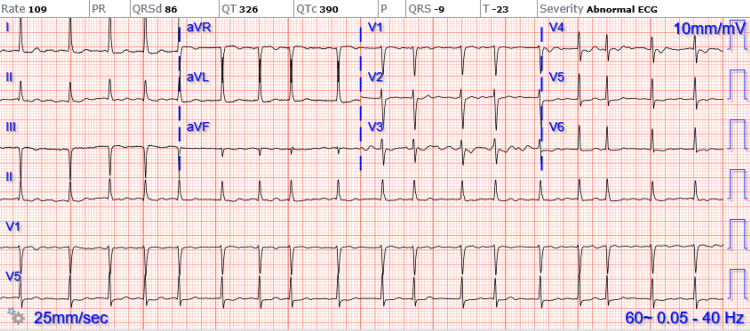
Electrocardiogram demonstrating atrial fibrillation without rapid ventricular response.

The patient’s initial vital signs included blood pressure of 153/109 mm Hg, heart rate of 111 beats per minute, respiratory rate of 25 breaths per minute, oxygen saturation of 100% on room air, and oral temperature of 99.1 degrees Fahrenheit. Physical examination revealed a 2 cm hematoma on the left forehead without active bleeding, though the head, neck, eyes, and ears were otherwise unremarkable. A soft, nontender abdomen and an irregular tachycardic heart rate were noted. Examination of the back revealed no spinal deformities, but she had diminished rectal tone and 0/5 strength in the bilateral lower extremities with intact sensation and 2+ patellar reflexes bilaterally. The clinician also noted cool lower extremities without dorsalis pedis or posterior tibialis pulses on Doppler examination. Electrocardiogram demonstrated atrial fibrillation without rapid ventricular response (Figure 1).

**Figure 2:**
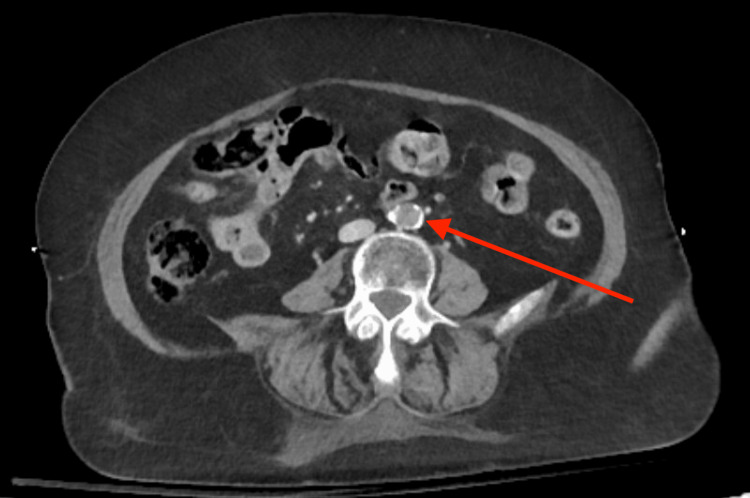
Computed tomography with angiography of the abdomen and pelvis on axial slice demonstrating the distal aorta occluded just proximal to the bifurcation. The red arrow denotes a 3-cm distal aortic plaque/thrombus.

**Figure 3:**
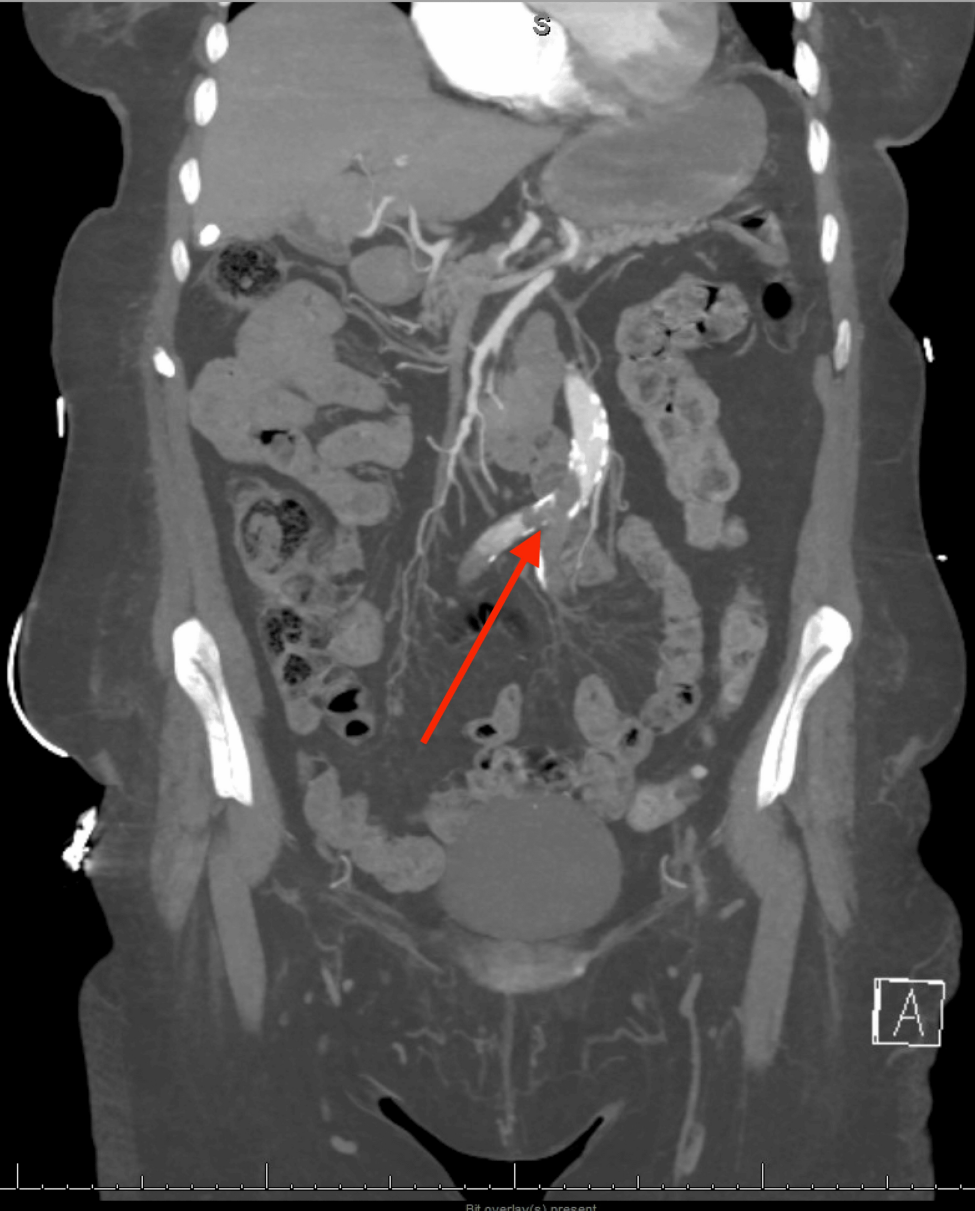
Similarly, computed tomography angiography of the abdomen on a coronal slice redemonstrated the 3 cm distal aortic occlusion, demonstrating the distal aorta occluded just above the bifurcation over a 3 cm segment by thrombus/plaque and extending into bilateral proximal common iliac arteries with distal reconstitution. The red arrow denotes a 3-cm distal aortic occlusion from plaque/thrombus.

Given the findings on examination, laboratory testing and imaging were obtained, to include computed tomography (CT) of the head, cervical, thoracic, and lumbar spine, as well as CT angiography (CTA) of the chest, abdomen, and pelvis. Laboratory evaluation revealed a lactate of 4.8 mg/dL (normal lactate less than 2.0 mg/dL) and an appropriately elevated prothrombin time of 16.3 seconds (normal prothrombin time 11-13.5 seconds) secondary to apixaban. She had an unremarkable complete blood count, comprehensive metabolic panel, and venous blood gas with an additional CT of the head without intracranial bleeding. CTA revealed a 3 cm infrarenal aortic occlusion extending into the common iliac arteries bilaterally (Figures 2, 3).

**Figure 4:**
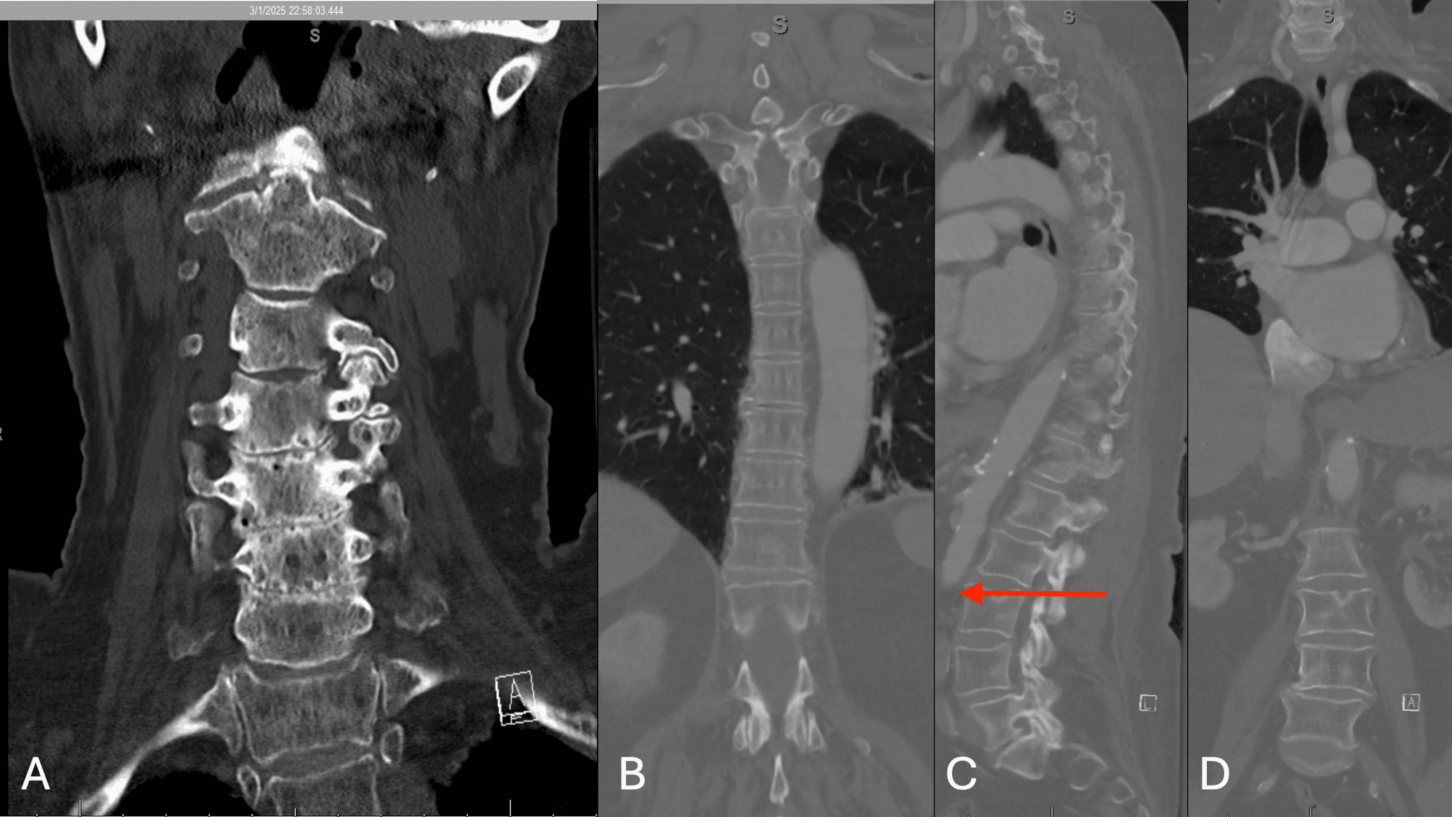
Computed tomography of the cervical, thoracic, and lumbar spine, without evidence of acute spinal fracture, dislocation, or injury. A: Coronal section of cervical spine computed tomography without acute spinal abnormality; B: Coronal section of thoracic spine computed tomography without acute spinal abnormality; C: Sagittal section of lumbar spine on computed tomography without acute spinal abnormality, though evidence of aortic occlusion (red arrow) better demonstrated on angiography in Figure 3; D: Coronal section of the lumbar spine on computed tomography without acute spinal abnormality.

Due to the concern for spinal trauma, CT of the cervical, thoracic, and lumbar spine was acquired, which demonstrated no acute spinal fracture, dislocation, or concerning lesion, though it did redemonstrate the aortic thrombus (Figures 4a-4c).

The emergency clinician emergently consulted the vascular surgeon, who performed operative thrombectomy of the aorta and external iliac arteries with prophylactic bilateral four-compartment lower extremity fasciotomies and lumbar drain placement; the prophylactic fasciotomy mitigates compartment syndrome, and the lumbar drain facilitates spinal perfusion. The patient’s hospital course was complicated by acute renal failure secondary to rhabdomyolysis and atrial fibrillation with rapid ventricular response. Her apixaban was restarted after an appropriate postoperative course. The patient regained 1/5 strength in the quadriceps bilaterally and 2/5 ankle and toe dorsiflexion and plantarflexion. She was discharged to a skilled nursing facility on hospital day 11.

## Discussion

Distal AAO represents a challenging but highly morbid diagnosis, with previous mortality rates near 100%, but they have improved to less than 20% with modern therapies if the condition is appropriately diagnosed [5-7]. AAO may present with a variety of symptoms, including lumbago, gastrointestinal issues, acute spinal cord pathology, and lower extremity weakness. As demonstrated in the above case, saddle embolus is more common in women and occurs in 21.3% of AAOs obstructing the bifurcation; as a result, patients will present with lower extremity pain and neurologic complaints [3,4]. Clinicians should evaluate for lower extremity pain, which occurs in 70.8% of patients, as well as sensory and motor deficits. These may occur secondary to both vascular compromise and spinal cord ischemia; 20.8% of patients will present with lower extremity paralysis, making this a challenging diagnosis to differentiate from a neurologic injury in the setting of trauma [1, 5]. Evaluation must include lower extremity neurologic, integumentary, and vascular examination to identify cyanosis, pallor, diminished or absent pulses, or neurologic deficits. The Rutherford classification system of limb ischemia may assist in clear communication with vascular consultants (Table 1) [5, 6].

**Table 1:** Rutherford classes of limb ischemia [5] Explicit permission for the use of this table has been obtained from the authors, Pelletier et al. 2024, and the publisher for the reproduction of this content.

Rutherford class	Sensory impairment	Motor impairment	Doppler signal
Class 1 (no immediate threat)	None	None	Arterial audible venous audible
Class 2a (marginally threatened)	Minimal	None	Arterial may be inaudible, venous audible
Class 2b (immediately threatened)	Involves forefoot, +/- pain at rest	Mild to moderate	Arterial absent, venous audible
Class 3 (irreversible ischemia)	Insensate	Severe, paralytic, rigor	Arterial absent, venous absent

An electrocardiogram may reveal new or pre-existing atrial fibrillation, suggesting a thromboembolic source [5]. While laboratory evaluation is limited, elevated lactate is associated with increased mortality, while coagulopathy and end-organ damage (e.g., elevated creatinine, liver function tests) may be present [7]. CTA of the chest, abdomen, and pelvis is the imaging modality of choice for rapid identification of both occlusion and any distal reconstitution, though point-of-care ultrasound can potentially identify aortic occlusion. However, data concerning ultrasound for the diagnosis of AAO are limited, and the reliability of ultrasound is based on operator skill and image quality [8,9]. While magnetic resonance imaging (MRI) can identify AAO, its temporal burden for this time-sensitive pathology renders MRI less useful in the ED setting [9].

Once identified, the ED management of AAO includes anticoagulation, symptomatic control (e.g., analgesia), and prompt vascular surgery consultation. Anticoagulation prevents further thrombus propagation or embolism expansion; specifically, unfractionated heparin is recommended by vascular surgical societies for its reversibility and relatively short half-life. [10,11]. While only conditionally recommended by vascular societies, prostacyclin analogues may improve perioperative mortality and decrease amputation rates, but should only be initiated in conjunction with vascular consultation [12, 13]. Acute limb ischemia is extremely painful, requiring analgesia with opioids as a first line of treatment, with ketamine as a supplemental analgesic [14,15]. ED clinicians should emergently consult the vascular surgery specialist or transfer to a center with vascular surgery capabilities for thrombolysis or endovascular or open procedures to definitively treat AAO as determined by both occlusion size and location, as well as the patient’s baseline status, comorbidities, previous vascular procedures, and anatomy. Endovascular techniques are significantly less invasive and have become the treatment strategy of choice if possible. Literature suggests they are responsible for a 9.7% decrease in mortality from 1994 to 2014 [3, 16].

Even with vascular intervention, AAO can beget a myriad of complications, with a third of patients experiencing pulmonary complications such as acute respiratory distress syndrome and a third experiencing renal complications, including acute kidney injury and potential need for renal replacement therapy [17]. Approximately 28% of patients will experience cardiac complications from increased afterload. Compartment syndrome can occur in 9% of patients managed with open vascular technique, though this has not been demonstrated in patients undergoing endovascular management. Revascularization by any technique does not guarantee restoration of neurologic status, and there is a risk of reperfusion syndrome even with appropriate vascular intervention, which presents with hemodynamic instability and multiorgan failure [1, 18].

## Conclusions

AAO carries significant morbidity and mortality due to distal tissue ischemia, and its presentation depends on the level and tissues affected. Distal or infrarenal AAO can be particularly difficult to diagnose, mimicking a significant number of other common conditions seen frequently in the ED. Occlusion can occur secondary to both thrombotic and embolic events, with tobacco use as the most common risk factor, though atrial fibrillation carries obvious thromboembolic risk as demonstrated in the above case. CTA is the diagnostic imaging modality of choice, while anticoagulation with unfractionated heparin should be initiated in the ED with vascular surgery consultation to facilitate definitive management such as thrombolysis, endovascular intervention, and/or open operative repair. Given the significant rate of morbidity in AAO, emergency clinicians must be aware of this serious pathology and consider it in their differential diagnosis.
